# Multiple factors regulate the expression of *sufCDSUB* in *Streptococcus mutans*

**DOI:** 10.3389/fcimb.2024.1499476

**Published:** 2024-11-27

**Authors:** Kassapa Ellepola, Lauren C. Guillot, Bradley Comeaux, Yiran Han, Jessica K. Kajfasz, Jacob P. Bitoun, Grace Spatafora, Jose A. Lemos, Zezhang T. Wen

**Affiliations:** ^1^ Department of Oral and Craniofacial Biology, School of Dentistry, Louisiana State University Health Sciences Center, New Orleans, LA, United States; ^2^ Department of Biology, Middlebury College, Middlebury, VT, United States; ^3^ Department of Oral Biology, School of Dentistry, University of Florida, Gainesville, FL, United States; ^4^ Department of Microbiology, Tulane University, New Orleans, LA, United States; ^5^ Department of Microbiology, Immunology and Parasitology, School of Medicine, Louisiana State University Health Sciences Center, New Orleans, LA, United States

**Keywords:** *Streptococcus mutans*, *sufCDSUB*, gene expression, PerR, CysR, SloR, SpxA, Dpr

## Abstract

**Introduction:**

The *sufCDSUB* gene cluster, encoding the sole iron-sulfur (Fe-S) cluster assembly system in *S. mutans*, was recently shown to be up-regulated in response to oxidative stressors and Fe limitation.

**Methods:**

In this study, luciferase reporter fusion assays, electrophoretic gel mobility shift assays (EMSA) and *in vitro* transcription assays (IVT) were used to dissect the *cis-* and *trans-*acting factors that regulate the expression of *sufCDSUB*.

**Results and discussion:**

Results showed deletion of *perR*, for the only Fur-family transcriptional regulator in *S. mutans*, resulted in >5-fold increases in luciferase activity under the control of the *sufCDSUB* promoter (P<0.01), as compared to the parent strain, UA159 when the reporter strains were grown in medium with no supplemental iron. Site-directed mutagenesis of a PerR-box in the promoter region led to elevation of the reporter activity by >1.6-fold (*P*<0.01). In an EMSA, recombinant PerR (rPerR) was shown to bind to the cognate *sufCDSUB* promoter leading to mobility retardation. On the other hand, the reporter activity was increased by >84-fold (P<0.001) in response to the addition of cysteine at 4 mM to the culture medium. Deletion of *cysR*, for a LysR-type of transcriptional regulator, led to reduction of the reporter activity by >11.6-fold (*P*<0.001). Addition of recombinant CysR (rCysR) to an EMSA caused mobility shift of the *sufCDSUB* promoter probe, indicative of rCysR-promoter interaction, and rCysR was shown to enhance *sufC* transcription under the direction of *sufCDSUB* promoter *in vitro*. These results suggest that multiple factors are involved in the regulation of *sufCDSUB* expression in response to environmental cues, including cysteine and Fe availability, consistent with the important role of *sufCDSUB* in *S. mutans* physiology.

## Introduction

*Streptococcus mutans*, a keystone microorganism in the development of human dental caries, is well known for its strong ability to colonize and accumulate on the tooth surface, especially in the presence of dietary sucrose, and utilize different sugars and produce lactic acid and other weak acids that leads to enamel demineralization and development of white spots and carious lesions (Lemos et al., 2019). The bacterium possesses at least three glycosyltransferase enzymes, GtfBC&D, which use sucrose, starch and other sugars as the substrate to produce extracellular polysaccharides (better known as glucans), including the water-insoluble alpha 3,2-linked polymers, playing a central role in the cariogenicity of the bacterium (Bowen and Koo, 2011). Multiple adhesins, including WapA, GtfB and the multi-functional adhesin SpaP (aka P1, AgI/II) interact with glycoprotein GP340 in the salivary pellicles to enhance the bacterial adhesion on the tooth surface, especially in the absence of sucrose (Lemos et al., 2019). *S. mutans* is also instrumental in the recruitment and establishment of other cariogenic bacteria such as *Candida albicans* and *Lactobacillus* spp. in the plaque microbiota (Gregoire et al., 2011; Wen et al., 2017), and under certain conditions, causing a shift in the highly dense and diverse plaque microbiota from homeostasis to dysbiosis, leading to the development of carious lesions.

Environmental conditions, such as the pH, temperature, oxygen tension and redox potential in the oral cavity are known to constantly fluctuate and often can be harsh and detrimental to the microbes in the environment. In addition, the oral cavity is often exposed to various antimicrobial agents, such as hydrogen peroxide, fluoride, and sodium dodecyl sulfate (SDS), from oral care products and as a result of localized and systemic drug therapy and/or preventative applications. *S. mutans* is also known for its ability to survive and adapt under different environmental conditions, such as low pH and oxidative stressors like hydrogen peroxide. Multiple pathways are utilized by the bacterium to cope with the environmental stressors, which include the chaperones DnaK and GroEL, two-component signal transduction systems CiaHR, VicRK, and LiaSR, the caseinolytic protease complex ClpCP, and others like surface associated protein BrpA (Lemos et al., 2019).

H_2_O_2_, superoxide anion (^•^O_2_^-^) and hydroxyl radicals (^•^OH) are among the reactive oxygen species (ROS) (Imlay, 2002; Miller and Britigan, 1997), which can cause serious damages to cellular macromolecules including proteins and DNA. Although H_2_O_2_ is relatively weak in toxicity compared with that of other ROS, in the presence of iron (Fe), it can be nonenzymatically converted into highly toxic hydroxyl radicals via Fenton reactions (H_2_O_2_ + Fe^+2^ → ^•^OH + -OH + Fe^+3^) (Enami et al., 2014). Bacterial species comprise multiple enzyme systems for the protection against ROS, such as catalase, superoxide dismutase (SOD), Dps-like protein (Dpr), alkyl hydroperoxide reductase (AhpCF), glutathione reductase, and thiol reductase (Fujishima et al., 2013). Although *S. mutans* lacks catalase, it contains AhpCF and Dpr that are primarily responsible for resistance to H_2_O_2_. AhpCF directly decompose H_2_O_2_, and Dpr inhibits the reaction of the Fenton pathway by capturing free Fe while SOD converts ^•^O_2_^-^ into H_2_O_2_ (Yamamoto et al., 2000; Fujishima et al., 2013).

The Fe-S clusters are known to be involved in electron transfer, sulfur mobilization, regulation and protein stabilization, catalysis and biochemical functions that are important for the biological activity of organisms including but not limited to their role as cofactors, substrate binding/activation and for iron storage (Lill, 2020, 2009). Due to the redox-active nature, Fe-S clusters are sensitive to cellular redox conditions, thus act as “molecular switches” for both transcriptional and translational regulation of gene expression (Sevilla et al., 2021). Many catalytic enzymes, including aconitase, 6-phosphogluconate dehydratase, fumarase A, NADH dehydrogenase, succinate dehydrogenase, and fumarate nitrate reductase (FNR), regulatory proteins, such as iron-sulfur cluster regulator (IscR) of *Escherichia coli* and SufR of *Cyanobacterium* spp (Vuorijoki et al., 2017), also contain Fe-S clusters directly regulating bacterial metabolism and gene expression (Fontecave et al., 2008).

Multiple Fe-S cluster biosynthesis and repair systems have been identified and described in different bacteria (Lill, 2009), including the SUF (sulfur mobilization) pathway that is encoded by the *sufCDSUB* operon, where *sufS* encodes a cysteine desulfurase. With cysteine serving as the sulfur source, cysteine desulfurase initiates the formation of Fe-S clusters by extracting the sulfur atom from cysteine generating a covalent persulfide intermediate and ferrying it to the Fe-S clusters along with alanine as a side product (Black and Dos Santos, 2015). This persulfide can then be conveyed directly to the synthesis machinery scaffold or via an intermediary carrier molecule (such as SufU or SufE) (Layer et al., 2007; Selbach et al., 2014). Unlike many other bacteria, *S. mutans* appears to possess only one Fe-S cluster machinery in SufCDSUB (Ellepola et al., 2021). Our recent studies by allelic exchange mutagenesis showed that SUF deficiency in *S. mutans* causes major defects in various cellular processes, but unlike any other bacteria that have been studied, the mutant lacking SUF is viable. Relative to the wild-type, UA159, mutants lacking *sufCDSUB* displayed major growth defects in an environment with low pH, oxidative and nitrosative stressors, and in the absence of certain amino acids such as leucine, isoleucine, and glutamate/glutamine whose biosynthesis require Fe-S clusters. Relative to the wild-type, mutants lacking *sufCDSUB* also had a significantly reduced survival rate following incubation at a low pH environment or under hydrogen peroxide challenge. Besides, the *sufCDSUB* mutant also tended to form aggregates and accumulated significantly less biofilms, especially during growth in medium with glucose. When analyzed using luciferase as a reporter, the expression of *sufCDSUB* was shown to be up-regulated in response to oxidative stresses. More than 5-fold increases were reported when the reporter strain was transferred from regular medium to medium with iron-limitation as well as medium with inclusion of an iron chelating agent.

Metalloregulatory proteins are essential for bacterial survival and pathogenesis due to their essential role in intracellular metal ion homeostasis. Among them is the ferric uptake repressor (Fur), a transcription factor which utilizes Fe^2+^ as a corepressor, thus involved in iron acquisition and storage, and regulates the production of some pathogenicity factors, the acid shock response, chemotaxis, and oxidative stress defenses in bacteria (Troxell and Hassan, 2013; Touati, 2000; Hantke, 2001). In *Bacillus subtilis* and certain other bacteria, Fur has been identified as a multifunctional protein that can also function as an iron-dependent activator (Pinochet-Barros and Helmann, 2020). The PerR (a Fur homologue) protein found in streptococci and several other bacteria with its peroxide-sensing ability is known to be associated with resistance to oxidative stresses (Brenot et al., 2005; Mongkolsuk and Helmann, 2002; Zhang et al., 2012). PerR in *S. mutans*, the sole Fur family protein encoded by SMU.593, regulates oxidative stress tolerance response when bound to a metal cofactor that, depending on the bacterial species, can be iron, manganese, or both (Ruxin et al., 2021; Lee and Helmann, 2006, 2007). SloR is also a metal-dependent transcription repressor, which regulates the expression of metal transporter SloABC (Spatafora et al., 2015). A recent study by Ruxin et al. has shown that SloR is regulated by repressor PerR, and together with PerR, plays essential roles in ion homeostasis and fitness of *S. mutans* (Ruxin et al., 2021).

Our recent studies have shown that *sufCDSUB* in *S. mutans* is regulated in response to environmental cues and regions in the *sufCDSUB* promoter as potential binding sites for PerR can be identified. This study is designed to further investigate how the expression of sufCDSUB is regulated by PerR and other regulatory factors. Results have shown that deletion of *perR* resulted in an increase of reporter activity by >5-fold, especially when grown under Fe-limitation, compared to the wild-type, but displayed no effect when growing in the presence of oxidative stressors. Unlike *perR*, deletion of *cysR* (SMU.852), encoding a LysR-type transcription regulator, led to >11-fold reduction of luciferase reporter activity. PCR-based deletions and point mutations, along with electrophoretic gel mobility shift assays (EMSA) and *in vitro* transcription (IVT) assays were used to further investigate how PerR and CysR regulate *sufCDSUB* expression. In addition, investigative proteomics was also used to elucidate the scope of the PerR-mediated regulation in *S. mutans.*

## Materials and methods

### Bacterial strains and cultivation

Bacterial strains and plasmids used in this study are listed in **Table 1**. *S. mutans* strains were maintained in brain heart infusion (BHI) medium. For biofilm formation, *S. mutans* was grown in modified biofilm medium (BM) with glucose (20 mM, BMG), sucrose (20 mM, BMS), or glucose (18 mM) plus sucrose (2 mM) as supplemental carbon and energy sources (BMGS) (Bitoun et al., 2012c). All solid media were prepared similarly with inclusion of Bacto agar (Difco Laboratories, Franklin Lakes, NJ) at the level of 1.5% (w/v). When needed, erythromycin (Erm, 10 µg/ml), kanamycin (Kan, 1 mg/ml), and/or spectinomycin (Spc, 1 mg/ml) were added. Unless otherwise stated, cells were grown at 37°C in an aerobic environment with 5% CO_2_. All *E. coli* strains were grown in Luria Bertani medium at 37°C aerobically, with or without inclusion of kanamycin (40 µg/ml), ampicillin (100 µg/ml), spectinomycin (100 µg/ml), and/or erythromycin (300 µg/ml).

**Table 1:** Bacterial strains and plasmids used in this study.

Strains/Plasmid	Major genotypes and phenotypes	References
*S. mutans* UA159	wild-type (ATCC 700610)	ATCC
*S. mutans* TW487	UA159 with *perR* deleted and replaced with *kan^r^*, Kan^r^	This study
*S. mutans* TW494	UA159 with *spxA1* deleted and replaced with *kan^r^*, Kan^r^	This study
*S. mutans* TW502	UA159 with inactivation of *SMU.1051* via insertion of *spc^r^*, Spc^r^	This study
*S. mutans* TW503	UA159 with *SMU.841* deleted and replaced with *spc^r^*, Spc^r^	This study
*S. mutans* JL13	UA159 with *spxA2* deleted and replaced with *erm^r^*, Erm^r^	(Kajfasz et al., 2015)
*S. mutans* TW505	UA159 with *dpr* deleted and replaced with *kan^r^*, Kan^r^	This study
*S. mutans* TW504	UA159 with *sloR* deleted and replaced with *kan^r^*, Kan^r^	This study
*S. mutans* GMS1386	UA159 with *perR* and *sloR* deleted and replaced with *kan^r^* and *erm^r^*, respectively, Kan^r^ and Erm^r^	(Ruxin et al., 2021)
*S. mutans* TW660	UA159 with *cysR* deleted and replaced with *kan^r^*, Kan^r^	This study
*E. coli/pET::spxA2*	BL21 (DE3)/pET::*spxA2*, Ap^r^	(Kajfasz et al., 2015)
*E. coli* M15*/*pQE30:*perR*	*E. coli* M13/pQE30:*perR*, Ap^r^, Kan^r^	This study
*E. coli* M15*/*pQE30:*cysR*	*E. coli* M13/pQE30:*cysR*, Ap^r^, Kan^r^	This study
pFW11	Integration vector with a promoterless luciferase reporter, Spc^r^	(Kreth et al., 2005)
Psuf	pFW11 fused with *suf* promoter, Spc^r^	(Ellepola et al., 2021)
PerRm	pFW11 fused with *suf* promoter with perR-box mutations, Spc^r^	This study
dBox1	pFW11 fused with *suf* promoter with Fur-box1 deletion, Spc^r^	This study
dBox2	pFW11 fused with *suf* promoter with Fur-box2 deletion, Spc^r^	This study
PgyrA	pFW11 fused with *gyrA* promoter, Spc^r^	(Ellepola et al., 2021)
Pldh	pFW11 fused with *ldh* promoter, Spc^r^	(Bitoun et al., 2012a)

*kan^r^*, *erm^r^, ap^r^* and *spc^r^* for non-polar kanamycin resistance (Kan^r^), non-polar erythromycin resistance (Erm^r^), ampicillin resistance (Ap^r^) and non-polar spectinomycin resistance (Spc^r^), respectively.

### DNA manipulation and mutant construction

Standard recombinant DNA procedures were used (Lau et al., 2002; Bitoun et al., 2013). All restriction and modifying enzymes were purchased from Invitrogen (Carlsbad, CA) or New England Biolabs (Ipswich, MA) and used as recommended by the suppliers. All primers (**Supplementary Table 1**) were synthesized by Integrated DNA Technologies, Inc. (Iowa City, IA). A *perR* deletional mutant was constructed using our well-established PCR-Ligation-Mutation protocol (Burne et al., 2011). Briefly, the 5’ and 3’ region of *perR* were amplified using high fidelity DNA polymerase Q5 (NEB Bioloabs) using gene-specific primers (**Supplementary Table 1**), and following proper restriction enzyme digestions, the flanking fragments were ligated with a non-polar spectinomycin resistant marker. Then, the ligation mix was used to transform *S. mutans* with inclusion of the competence stimulating peptide, and mutants lacking *perR* was isolated from plates with spectinomycin. Isolated mutants were verified by PCR and Sanger sequencing with gene-specific primers (**Supplementary Table 1**). *S. mutans* strains with deletion of *dpr*, *sloR*, and *cysR* were generated using similar strategies.

### Promoter mapping and luciferase reporter fusion assays

The transcription start sites were determined using the Template Switching RT Enzyme Mix (NEB #M0466) by following the 5’ RACE protocol (NEBLABS, Ipswich, MA). For total RNA, wild-type *S. mutans* UA159 cells were harvested at middle exponential phase (OD_600_ ≈ 0.3) and immediately treated with RNAProtect^®^ (Qiagen, Inc). Total RNAs were extracted using the hot phenol method and purified using RNAeasy kit (Qiagen, Inc.), and DNase I treatment including an in-Column DNase I treatment was used to clean up the residual genomic DNA. 1 µg total RNA was used in the template switching reverse transcription, and the resulting cDNA with inclusion of a universal sequence attached to its 3’ were amplified by PCR using high fidelity DNA polymerase Q5, and the transcription initiation site was determined by Sanger sequencing.

To analyze the regulation of *suf* expression, a promoterless luciferase gene (*luc*) was used as a reporter as previously described (Kreth et al., 2005). Briefly, the cognate *suf* promoter region and its derivatives generated by PCR-based deletions and/or site-directed mutations were amplified using high fidelity DNA polymerase Q5 (NEB Biolabs), and following proper restriction digestions, cloned directly in front of the promoterless *luc* gene in integration vector pFW11-*luc* (Kreth et al., 2005), which also contains a Shine-Dalgarno sequence optimized for group A streptococci (Podbielski et al., 1999). Following confirmation of the cloned element by sequencing, the resulting constructs were introduced into *S. mutans* UA159 and its selected derivatives such as the *perR* deletion mutant, Δ*perR*, and the expression of *suf* were analyzed using luciferase assay by following the protocol of Podbielski (Kreth et al., 2005; Bitoun et al., 2012a, 2012). For controls, pFW11 with promoterless reporter and a luciferase reporter fused with the gyrase promoter (P*gyr*) were used (Ellepola et al., 2021).

### Protein expression and purification

For expression and purification of recombinant PerR (rPerR), recombinant CysR (SMU.852), and recombinant Sigma factor (rSigA), the respective coding regions were amplified by PCR with gene-specific primers (**Supplementary Table 1**) using high fidelity DNA polymerase Q5 (NEB Biolabs), and cloned in pQE30 (Qiagen, Inc.). Following Sanger sequencing to confirm accuracy of the cloned DNA, the resulting constructs were transformed into *E. coli* M15, and PerR, CysR and SigA expression were induced by addition of IPTG at 0.5 mM (final conc.), and the His-tagged rPerR, rCysR and rSigA were purified using nickel resins (Fisher Scientific) under native conditions similarly as described previously (Bitoun et al., 2012c). The purified proteins were then applied to Amicon Ultra 3 Centrifugal Filters (3 kDa MWCO, Millipore) for desalting and re-constituted in 20 mM Tris, pH 7.6 plus 20% glycerol. For PerR, 10 mM EDTA was also included in the elution buffer, desalting and exchange buffers (Lee and Helmann, 2006).

For *in vitro* transcription assays, crude native *S. mutans* RNA Polymerase (RNAP) was obtained based upon the methods described by Seepersaud et al. (Seepersaud et al., 2006) and Galvão et al. (Galvao et al., 2015). Briefly, *S. mutans* cells of mid-exponential phase were harvested by centrifugation, resuspended and incubated in protoplast preparation buffer at 37°C for 90 minutes. Protoplasts were then pelleted and resuspended in lysis buffer, lysed by glass bead beating and, the supernatants were applied to Affi-Gel heparin resin (Bio-Rad) and eluted with a NaCl gradient of 0.1 to 1 M. Fractions containing visible subunits were pooled and applied to Macro-Prep High-Q ion exchange resin (Bio-Rad) and eluted with a gradient of 0.1–0.8 M NaCl. The desired fractions were again pooled and dialyzed.

### Protein-DNA interactions

To assess if and how transcriptional factor PerR interacts with the *sufCDSUB* promoter, EMSA was employed similarly as described previously (Bitoun et al., 2012a). Briefly, the intact promoter region and its derivatives with deletions and/or site-directed mutations were amplified by PCR using Q5 DNA polymerase with the primers listed in **Supplementary Table 1**. The PCR products, with biotinylation at the N-terminus or without (for cold-competition control), were incubated with increasing amounts of rPerR in the reaction buffer at room temperature (Bitoun et al., 2012a). The reaction mixtures were separated on a 5% polyacrylamide gel under native conditions, and DNA mobility shift was visualized via LightShift Chemiluminescent EMSA Kit by following procedures recommended by the manufacturer (Pierce, Rockford, IL) or by SYBR Gold staining (Invitrogen, CA) and a GelDoc Go Imaging system (BioRad).

### IVT assays

*In vitro* transcription assay was performed by following the protocols of Dou et al (Dou et al., 2021) and Galvao et al (Galvao et al., 2015) using native *S. mutans* RNAP and rSigA. In some experiments, *E. coli* RNA polymerase holoenzyme (M0251S, NEB Biolabs) (2 units) was also used. Briefly, a DNA fragment carrying the *sufCDSUB* promoter plus 834 bp of the coding sequence was amplified by Q5 DNA polymerase and purified using Qiagen PCR purification kit (Qiagen, Inc.). For IVT, ~100 fM of the DNA template, 25 nM of *S. mutans* RNAP, and 25 nM of rSigA were incubated with and without rPerR or rCysR at different µM concentrations in buffer containing 20 mM Tris-HCl (pH 7.5), 50 mM KCl, 5 mM MgCl_2_, 0.1 mM DTT, 0.01% Triton X-100, 5 mM of NTP mix at 37°C for 1 hour. The resulting transcripts were analyzed using 1.4% denaturing agarose gel and visualized by SYBR Gold staining (InVitrogen, CA) similarly as described above.

### Proteomics analysis

Proteomics analysis was carried out similarly as described previously (Wen et al., 2006; Yue and Guidry, 2019). Briefly, cultures were grown in regular BHI until mid-exponential phase (OD_600nm_~0.4) and homologized in Tris-HCl buffer, 50 mM pH 7.4 containing 10% glycerol using a glass bead beater as described (Wen et al., 2006). Following protein concentration estimation by BCA assay (Pierce, ThermoFisher), 100 µg of each protein sample was digested with trypsin, and labeled using a Tandem Mass Tag (TMT) 10-plex Reagent set (Pierce, ThermoFisher). HPLC-MS/MS analysis was done in four technical replicates with a false discovery rate (FDR) of < 1% at the LSUHSC Proteomics Core by following established protocols.

### Statistical analysis

At least three biological and two technical replicates each were used for all the experiments. Quantitative data were analyzed using paired Student *t* test. A *P* value of 0.05 or less is considered statistically significant.

## Results

### Multiple *cis-*acting elements, including a Fur-box and a PerR-box, are involved in *sufCDSUB* expression

**Figure 1:**
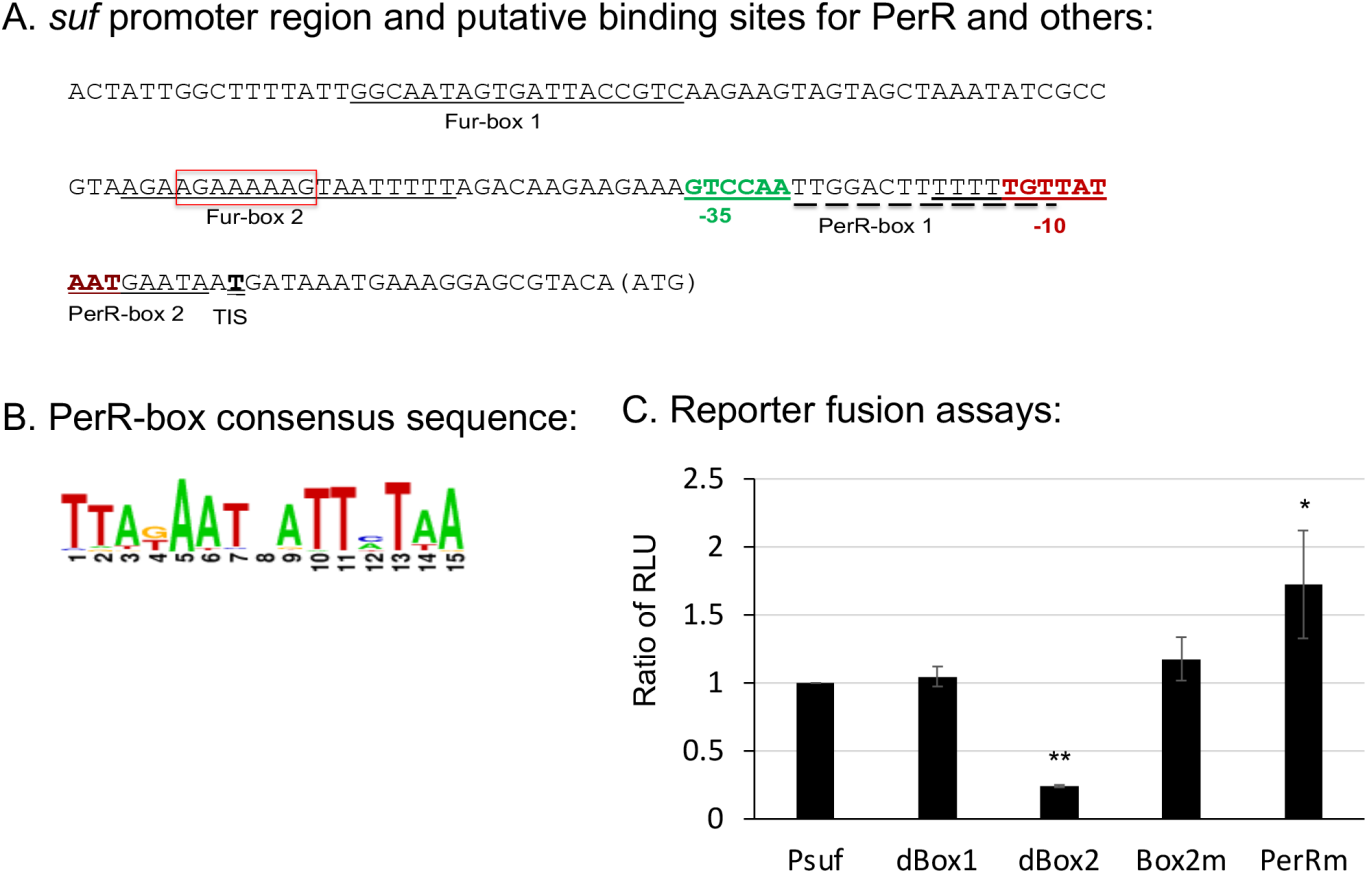
The *sufCDSUB* promoter region and the roles of different major elements in *sufCDSUB* expression. **(A)** Schematic diagram of the *sufCDSUB* promoter region with extended -10 region and -35 region in red and green, respectively, and the red box indicating the sequences deleted in Fur-box 2 (dBox2); **(B)** Consensus of PerR-boxes; **(C)** The effects of deletion of the conserved Fur-box 1 (dBox1), Fur-box 2 (dBox2) and site-directed mutations in Fur-box 2 (Box2m) and PerR-box 1 (PerRm) on luciferase reporter activity, as compared to the wild-type, Psuf **(C)**. Data are expressed as ratios of the relative light units (RLU) of the respective mutant over the wild-type. *, ** P<0.05 and 0.01 vs Psuf via Student’s *t* test.

First, rapid amplification of cDNA ends (5’RACE) was employed to determine the transcription initiation site of *sufCDSUB*, and it was done using total RNA isolated from mid-exponential phase cultures grown in regular BHI broth. The results showed that under these conditions, the transcription initiation site of the *sufCDSUB* operon was consistently mapped at nucleotide 22 from the translation initiation site ATG (**Figure 1A**). The promoter region was then subjected to virtual footprinting analysis (https://www.prodoric.de/vfp/), and multiple regions were identified with potential roles in *sufCDSUB* regulation, which include two regions, termed Fur-box1 and Fur-box2 with high similarity to the binding site of Fur (consensus: GGCAATAGTGATTACCGTC) and two regions with similarity to the PerR-binding site (consensus: TTATAATnATTATAA) (**Figure 1B**). To further examine the role of these respective regions in *sufCDSUB* expression, PCR-based mutations and deletions of the *sufCDSUB* promoter were made using primers listed in **Supplementary Table 1**, and the resulting constructs were directly cloned in front of the promoterless luciferase reporter in pFW11 (Kreth et al., 2005; Bitoun et al., 2012a). As shown in **Figure 1C**, mutations of the PerR-box1 from TTGGACTTTTTTTGTT to aaGatCaTTTTTTGTT (low case indicated mutated nucleotides) in PerRm resulted in elevation of the reporter activity by 1.65-fold (*P*<0.05), compared to the intact promoter-reporter fusion Psuf, indicative of a role as a potential binding site of a transcriptional repressor. Site-directed mutation of Fur-box 2 (AGAAGAAAAAGTAATTTTT, Box2m), where nt GAAAAAGT were changed to ctttttgt, showed no changes in the luciferase activity compared to the intact promoter-reporter fusion. Interestingly, deletion of AGAAAAAG in Fur-box 2 (dBox2, shown in red-box) led to >4.23-fold reduction of the reporter activity, while no significant effect was observed with the deletion of Fur-box1 (dBox1) (**Figure 1C**). The results suggest that Fur-box2 is likely a binding site for a positive regulator.

### PerR is a transcription repressor of *sufCDSUB*

**Figure 2:**
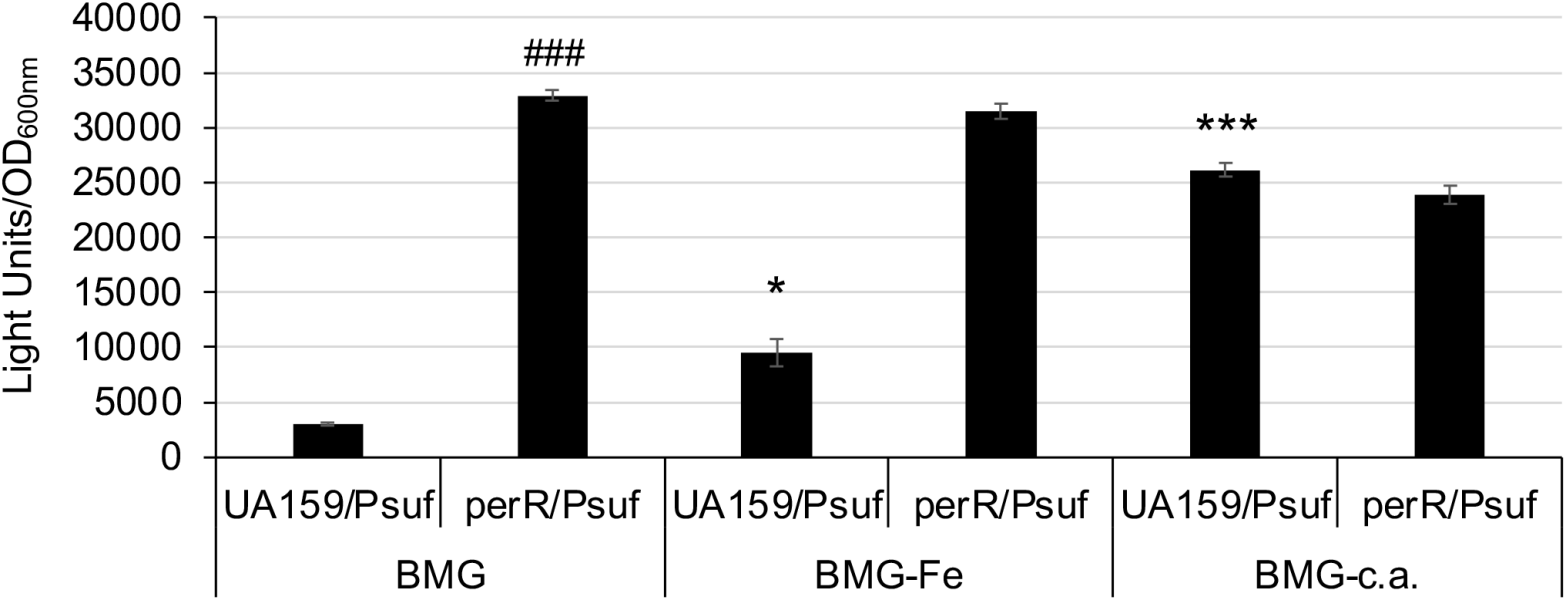
Luciferase reporter analysis in response to Fe-limitation. *S. mutans* UA159 and its *perR* mutant were transferred from regular BHI broth medium to semi-defined biofilm medium plus glucose with (BMG) and without inclusion of FeCl_2_ (BMG-Fe) and with addition of 2,2’-pyridyl, a chelating agent (BMG-c.a.) at 50 µM. *, ***, P<0.05 and 0.001 vs UA159/Psuf in BMG, respectively; ###, P<0.001 vs UA159/Psuf, via Student *t-*test.

*S. mutans* possesses a PerR (SMU.593) (Kajfasz et al., 2021), the sole Fur family of metal ion-dependent transcriptional regulator that modulates the expression of target genes by sensing and responding to oxidative stress (Bsat et al., 1998). To assess the role of PerR in *sufCDSUB* regulation, an allelic exchange mutant of *perR* was constructed with a major portion of the coding region replaced by a non-polar kanamycin resistance marker (Burne et al., 2011). As expected, deletion of *perR* led to de-repression of *sufCDSUB* expression, especially when grown in Fe-rich biofilm medium. Relative to the wild-type, the *perR* deletion mutant displayed an increase of >2-fold in luciferase reporter activity when the cultures were grown in regular BHI (*P*<0.05) (**Figure 2**). Such differences were increased to as much as 10-fold compared to the wild-type, when the reporter strains were transferred from BHI to semi-defined biofilm medium. Consistent with our recent studies (Ellepola et al., 2021), luciferase reporter activity of the wild-type UA159 was increased by >5.3(±1.1)-fold when the BHI-grown overnight cultures were transferred to semi-defined biofilm medium with glucose (BMG), especially when FeCl_3_ was omitted from the medium recipe (BMG-Fe) (**Figure 2**). The highest reporter activity of the wild-type was measured when the cultures were grown in BMG medium with inclusion of 2,2’-pyridyl (ACROS Organics, #366-18-7), an iron chelating agent at 50 µM. However, no such effects were measured when overnight cultures were also grown in BMG or BMG-Fe. Like the wild-type, the luciferase reporter activity of the *perR* mutant was also increased when it was transferred from regular BHI to BMG. Compared to the wild-type, the reporter activity of the *perR* mutant was increased by >10-fold of the wild-type (*P<*0.001). However, unlike the wild-type, no significant differences in luciferase reporter activities were measured between the *perR* mutant when grown in BMG and those in BMG-Fe and BMG plus 2,2’-pyridyl at 50 µM (**Figure 2**). These results further suggest PerR plays a major role as a repressor in *sufCDSUB* expression in response to Fe-availability in the culture medium, and consistent with other Fur regulators, it requires Fe as a co-factor.

### PerR interacts with the *sufCDSUB* promoter, influencing its transcription *in vitro*

**Figure 3:**
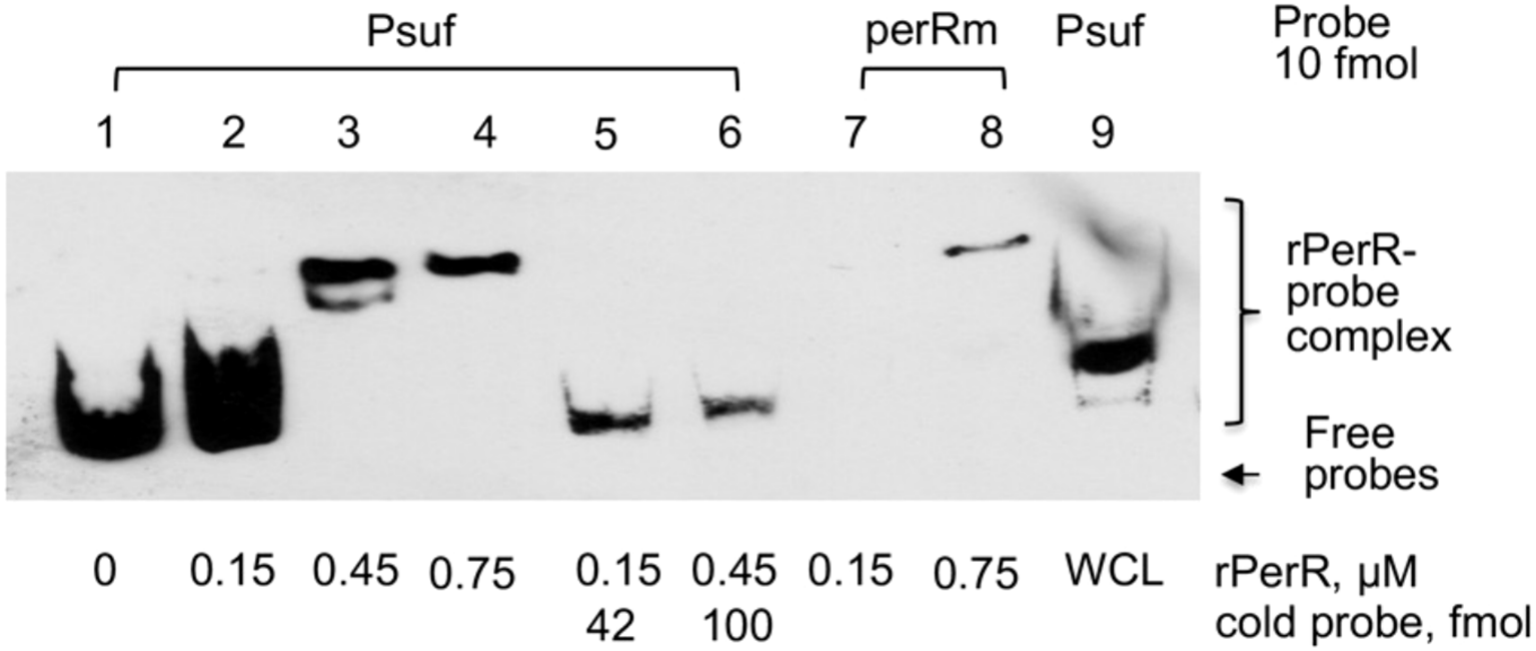
EMSA assays of PerR-*sufCDSUB* promoter interactions. Inclusion of rPerR resulted in mobility retardation of biotinylated *suf* promoter DNA probes (Psuf, ln 1-4), and such interaction was concentration-dependent as indicated. Addition of cold-probe led to release of biotinylated probes from the rPerR-Psuf complex (Psuf, ln 5&6). Similar effects were also observed when whole cell lysate of *S. mutans* UA159 (WCL) was used. However, mutations in the PerR-box 1 (perRm) showed no effect under the conditions studied.

To further examine if PerR interacts with the *sufCDSUB* promoter influencing its transcription, PerR was expressed in *E. coli* by isopropyl β-d-1-thiogalactopyranoside (IPTG) induction and purified using Ni^2+^ affinity columns, and EMSA assays were conducted using the purified rPerR. Results showed that inclusion of rPerR in the reaction mix resulted in mobility shift of the *sufCDSUB* promoter probe, and such activity was concentration dependent (**Figure 3**). The mobility shift was negated or reduced significantly when rPerR protein was first incubated with non-biotinylated promoter oligo. Reduced mobility shift was also observed when whole cell lysates of the wild-type *S. mutans* UA159 were used in the reaction. Interestingly, no major differences were observed in mobility shift when rPerR was mixed with oligo PerRm, which has the PerR-box1 mutated from TTGGACTTTTTTTGTT to aaGatCaTTTTTTGTT using PCR-based site-directed mutagenesis (see **Figure 1**). These results further suggest that PerR interacts with the *sufCDSUB* promoter, although the exact nucleotides that interact with PerR repressor remain unclear. In an IVT assay using native *S. mutans* RNA polymerase (RNAP) and recombinant Sigma factor 70 (rRpoD) with the whole promoter plus 834 bp of the coding sequence of *sufC*, a transcript of expected size was detected. However, inclusion of rPerR in the reaction mix only slightly reduced the amount of the transcripts, but no such effects were observed when recombinant LuxS protein or none was added as controls.

### PerR deficiency alters the expression of a number of genes including those in oxidative stress tolerance response

**Figure 4:**
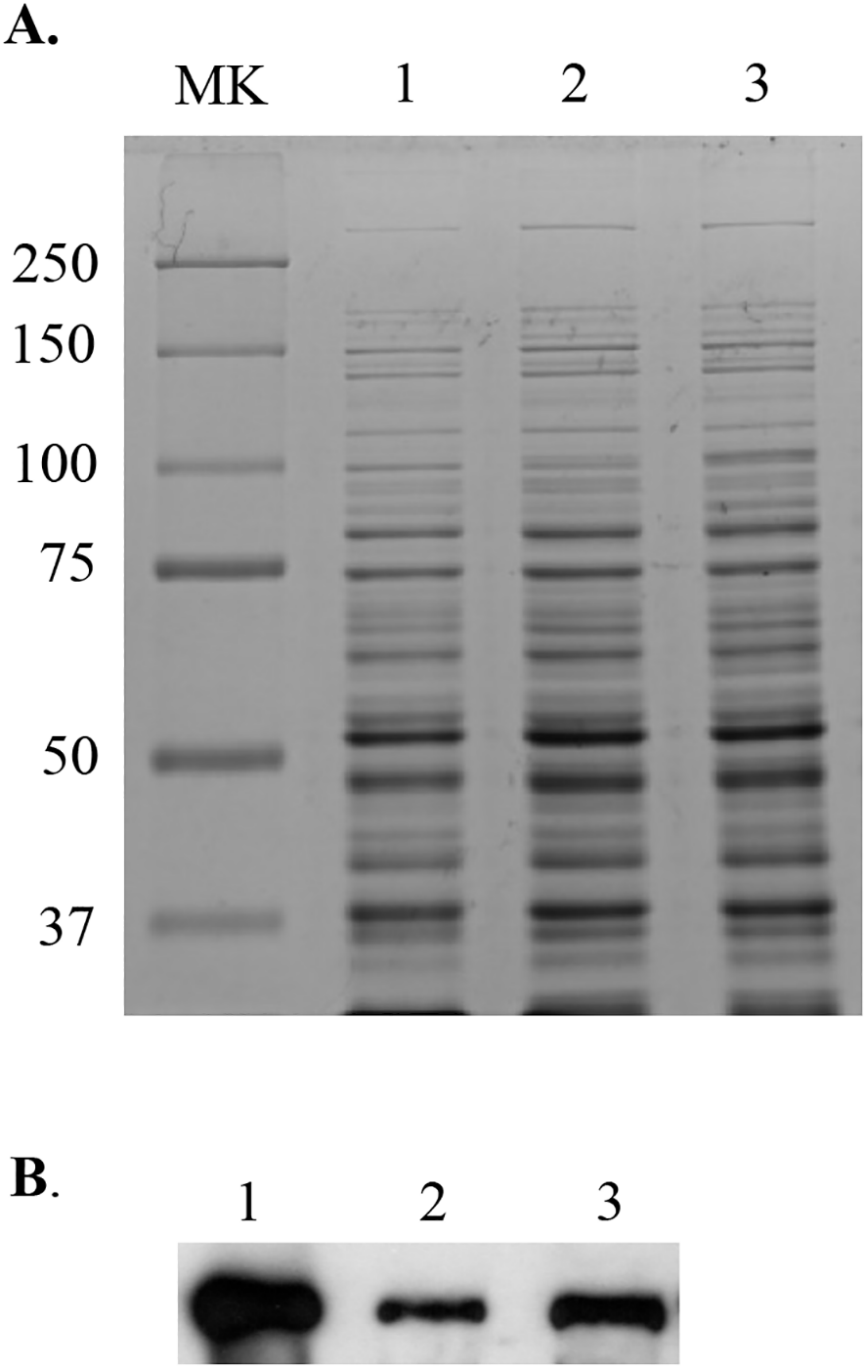
Western blot analysis of the *perR* mutant. Whole cell lysates (10 µg of total proteins) of *S. mutans* wild-type UA159 (lane 1) and its *perR* (lane 2) and *sufCDSUB* (lane 3) mutants were separated using 10% SDS-PAGE **(A)**, blotted to a PVDF membrane, and then probed with polyclonal antibodies against FruA **(B)**. MK, molecular weight marker in kilodalton.

A proteomics approach was also used to examine the protein profile of the *perR* mutant, and the results showed that when compared to the wild-type UA159, the expression of more than 35 proteins were altered as a result of *perR* deletion, including 17 up-regulated proteins and 18 down regulated (**Supplementary Table 2**). Among the up-regulated proteins were those with roles in oxidative stress tolerance responses including Dpr, thiol peroxidase Tpx, and putative metal transporter SMU.635, consistent with the recent RNA-seq studies by Kajfasz et al (Kajfasz et al., 2021). Interestingly, several enzymes of sugar transport and metabolism pathways were also found to be altered significantly, including down-expression of β-exo-fructanase FruA, a well-known virulence attributor, and up-expression of sucrose- and mannose-specific transporters (**Supplementary Table 2**). When whole cell lysates were separated by SDS-PAGE and probed in a Western blotting using polyclonal antibodies (a kind gift of Dr. RA Burne), the results further confirmed the reduction of FruA expression in the *perR* mutant as well as the *sufCDSUB* mutant (**Figure 4**), compared to the parent strain, UA159. However, it awaits further investigation if the expression of these genes is directly regulated by PerR.

### Dpr regulates oxidative stress indirectly influencing *sufCDSUB* expression

**Figure 5:**
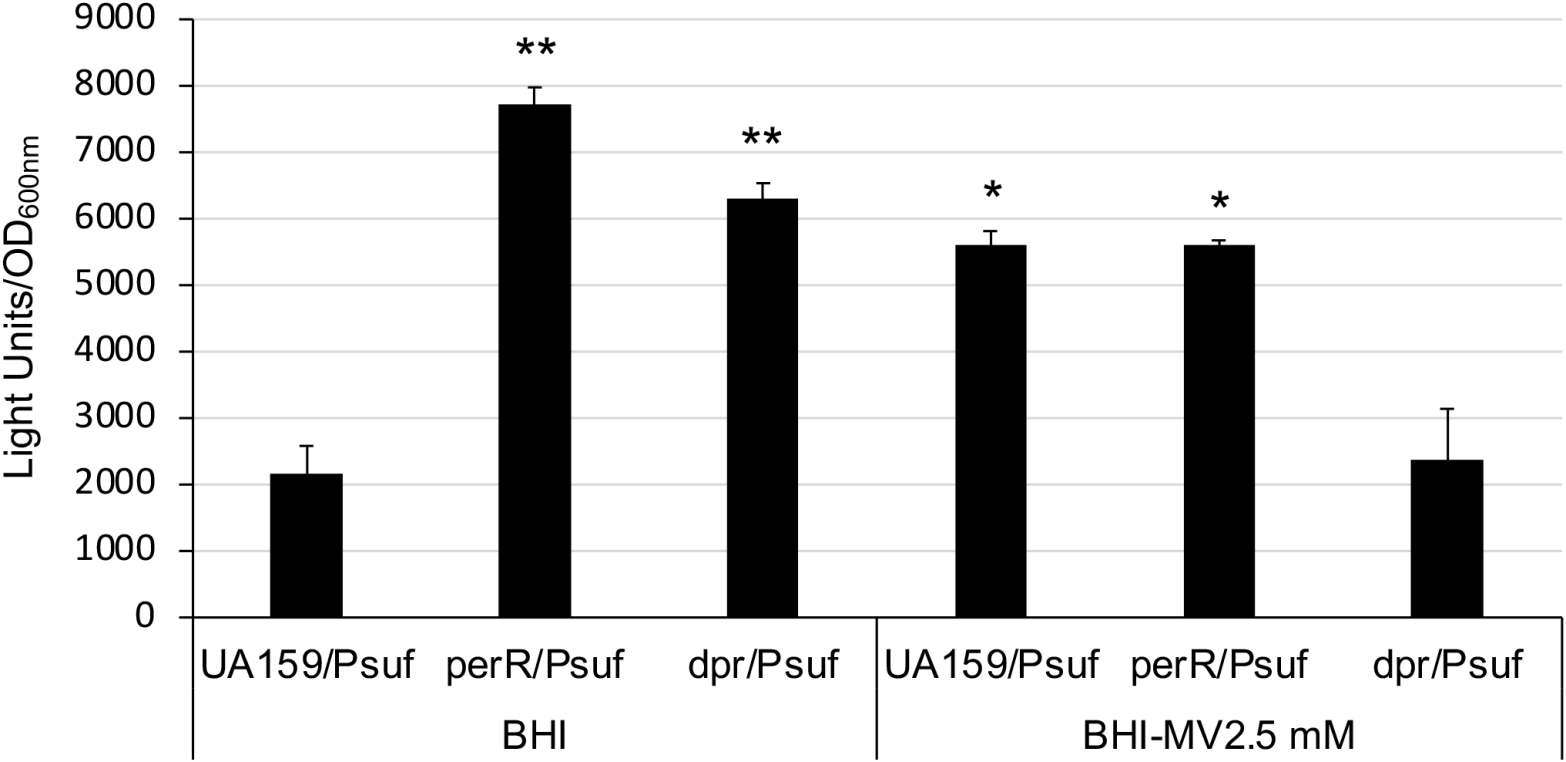
Luciferase reporter analysis of a *dpr* mutant. Overnight cultures of *S. mutans* UA159 and its *perR* and *dpr* mutants were washed thrice and then transferred to BHI broth with and without methyl viologen (MV) at 2.5 mM and let grow in an anaerobic box. Results showed the *dpr* mutant grew slowly than UA159 and the *perR* mutant, but similarly as UA159 and the *perR* mutant, could still launch oxidative stress responses and was featured with induction of luciferase reporter activity. However, unlike the parent strain and the *perR* mutant, no such activities were observed when it was grown in the presence of 2.5 mM MV. *, **, P<0.05 and 0.01, respectively, when compared to UA159/Psuf in BHI by Student *t-*test.

Our previous study showed that expression of the *sufCDSUB* operon is regulated in response to oxidative stressors (Ellepola et al., 2021). Relative to cultures grown in regular BHI, the luciferase reporter activity was increased by >2-fold in cultures growing in the presence of methyl viologen (at 2.5 mM) (**Figure 5**), a molecule that induces superoxide production inside the cells. Like the wild-type, luciferase reporter activity of the *perR* mutant was also increased following exposure to methyl viologen at 2.5 mM, but unlike growing in regular BHI, the differences between the wild-type and the *perR* mutant under the condition with 2.5 mM methyl viologen were not statistically significant (**Figure 5**). These results suggest that factors other than PerR are likely involved in the oxidative stress mediated regulation of *sufCDSUB*.

Dpr is known to as a major factor in resistance to reactive oxygen species. To test if Dpr mediated oxidative stress tolerance response influences *sufCDSUB* expression, an allelic exchange *dpr* mutant was constructed, and the impact of *dpr* deletion on *sufCDSUB* expression was analyzed using luciferase reporter fusion assays similarly as described above. Consistent with Ganguly et al (Ganguly et al., 2018), *dpr* deletion led to major reductions in growth rate and viability of the deficient mutant under aerobic conditions due to compromises in oxidative stress tolerance. Consistently, the luciferase reporter activity in the *dpr* mutant was increased, as compared to the wild-type, when the reporter strains were grown in an anaerobic box (**Figure 5**). However, no such effects were observed when methyl viologen at 2.5 mM was included in the growth medium especially under aerobic conditions, which again indicates a compromised oxidative stress tolerance response of the *dpr* mutant. Similar trends were also observed with the gyrase A promoter fusion as a reference. These results further suggest that oxidative stressors strongly influence the expression of *sufCDSUB*, and that Dpr-deficiency induces oxidative stresses of the deficient mutant, indirectly affecting *sufCDSUB* expression.

### Oxidative stress regulator SpxA2 positively regulates *sufCDSUB* expression

**Figure 6:**
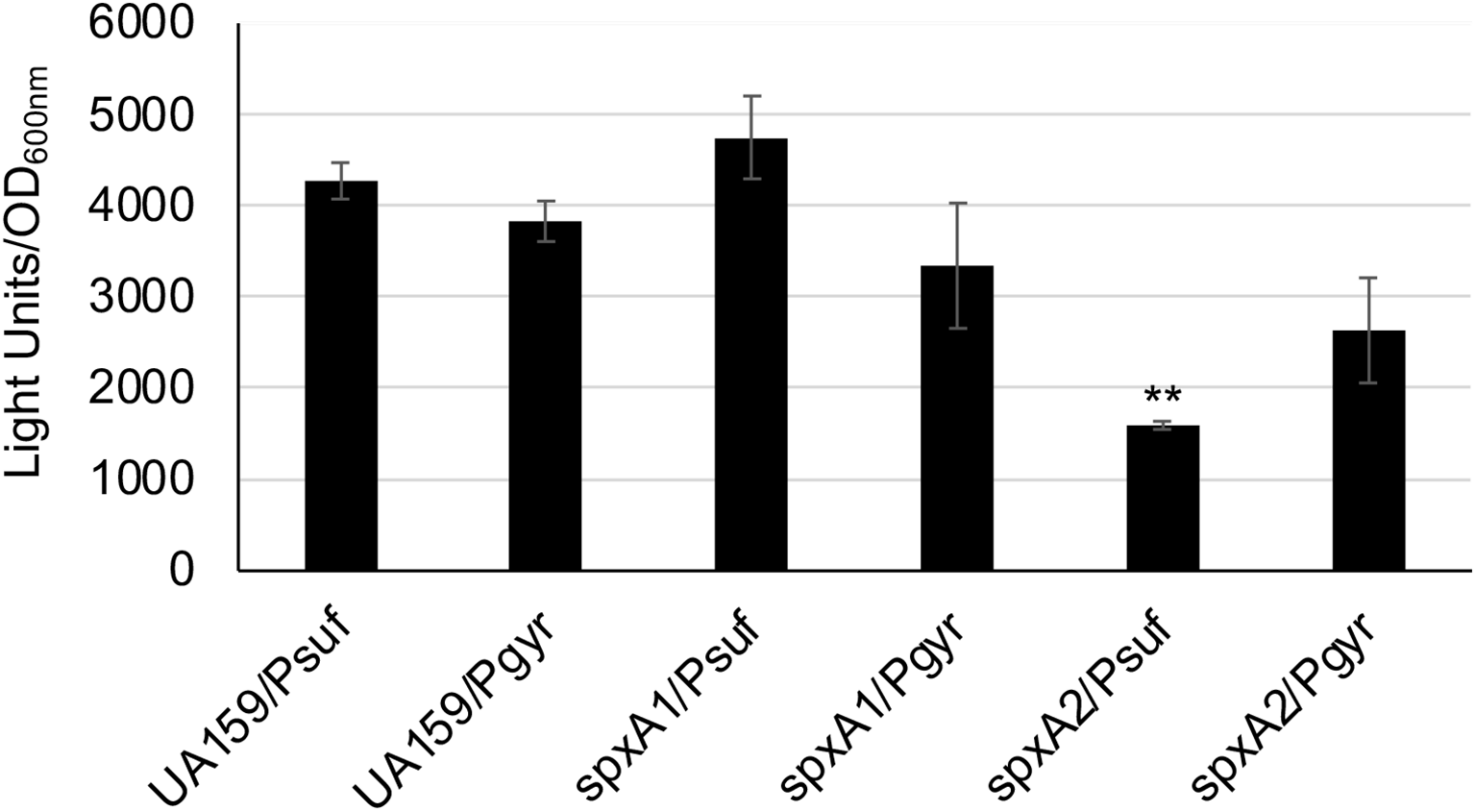
Luciferase reporter fusion analysis of the *spxA* mutants. *S. mutans* wild-type (UA159) and its *spxA1* (spxA1) *& spxA2* (spxA2) deficient mutants carrying the *suf* promoter-reporter fusion (Psuf) or the *gyrA* promoter-reporter fusion (Pgyr) as a reference were grown in BHI broth to mid-exponential phase (OD_600nm_ ~0.4), when luciferase activities were analyzed. **, *P*<0.01 vs UA159 under the same condition via Student *t-*test.

*S. mutans* possesses two Spx homologues, SpxA1 and SpxA2, which serve as the activator of genes involved in oxidative stress response (Galvao et al., 2015; Kajfasz et al., 2015). By RNA-seq analysis, Kajfasz et al (Kajfasz et al., 2017) found that members of the *sufCDSUB* operon were up-regulated in the wild-type following exposure to sub-lethal amount of hydrogen peroxide, but they were significantly reduced in *spxA1* mutant and more so in a *spxA1/spxA2* double mutant, compared to the wild-type, UA159. To further investigate how the SpxA regulators influence the expression of the SUF machinery, the *suf* promoter-reporter fusion construct was transformed into the allelic exchange *spxA1* and *spxA2* mutants, and the impact of *spxA* deletion on *sufCDSUB* expression was analyzed using luciferase reporter assays with the gyrase *gyrA* promoter PgyrA-reporter fusion serving as a reference. As compared to the wild-type, the luciferase reporter activities under the direction of P*suf* promoter were reduced by >2.8-fold (*P*<0.01) (**Figure 6**) in the *spxA2* mutant, which in part can also be attributed to the fact that the *spxA2* mutants grew more slowly as compared to the wild-type, UA159 (Galvao et al., 2015). No significant differences were observed between the *spxA1* mutant and the wild-type, UA159. The reference P*gyrA*-reporter fusion showed no significant change in expression under the conditions tested.

### SloR-deficiency indirectly influences *sufCDSUB* expression

**Figure 7:**
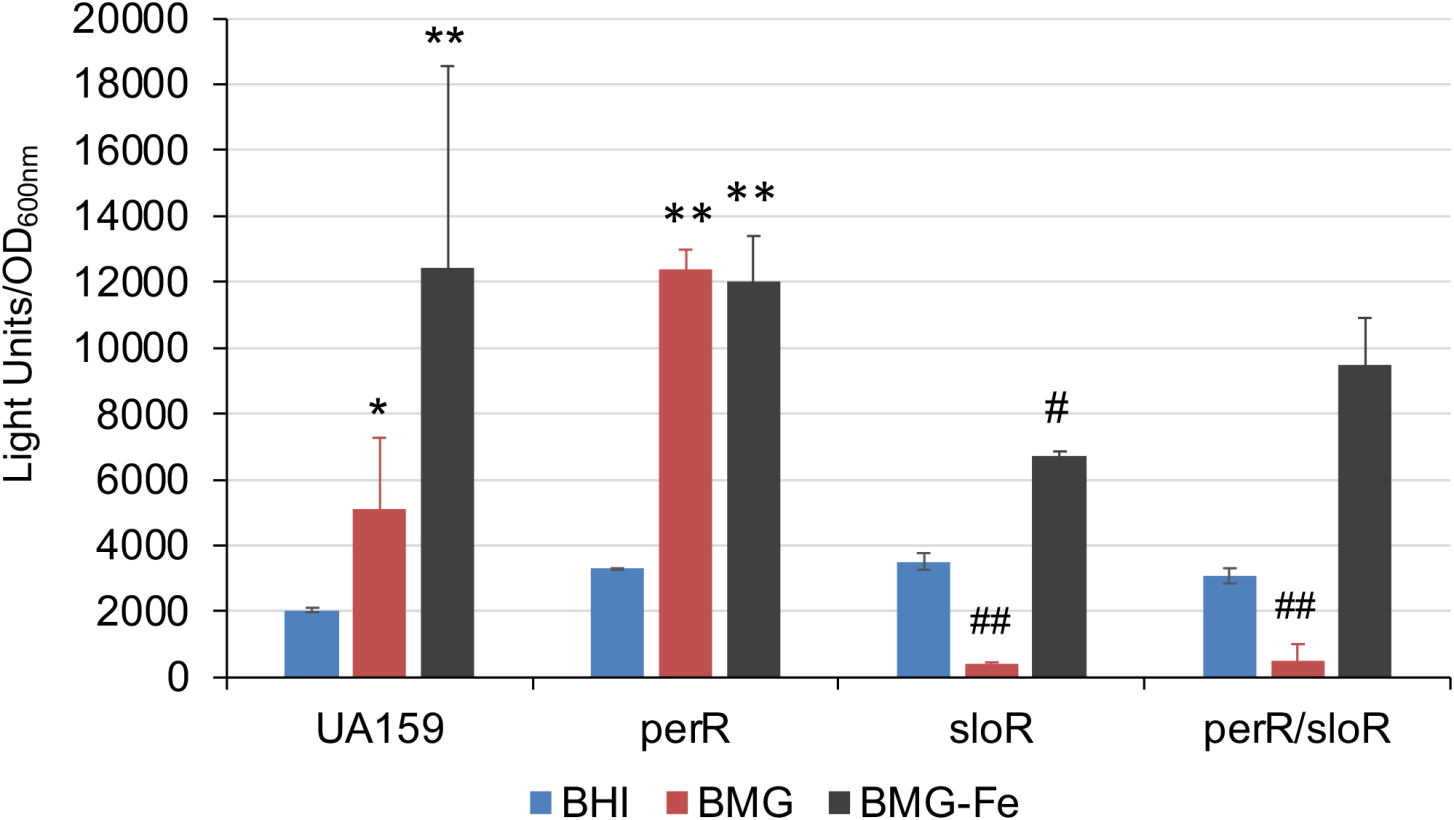
Luciferase reporter analysis of a *sloR* mutant and a *sloR/perR* mutant. *S. mutans* wild-type UA159 and its *sloR* and *sloR/perR* mutants were grown in BHI, BM plus glucose with (BMG) and without inclusion of FeCl_2_ (BMG-Fe). *, **, P<0.05 and 0.01, respectively, when compared to growth in BHI; #, ##, P<0.05 and 0.01 vs UA159 in BMG-Fe and BMG, respectively, while analyzed by Student *t* test.

SloR is known as a metal-dependent repressor of *sloABC* when Fe is plentiful in the growth environment (Rolerson et al., 2006). SloR also facilitates its own expression as a positive autoregulator (Monette et al., 2018). To investigate potential roles of SloR in *sufCDSUB* expression, an allelic exchange *sloR* (SMU.186) mutant was constructed and further analyzed, along with a *perR/sloR* double mutant, using luciferase reporter assays as described above. Relative to the parent strain, UA159 and the *perR* mutant, the *sloR* mutant displayed some aberrant growth phenotypes including extended lag phase (>24 hours) and aggregations when growing in regular BMG medium. However, both the *sloR* single and *sloR/perR* double mutant grew similarly well as the wild-type in BMG medium with omission of iron in the recipe (BMG-Fe) or BMG with inclusion of chelating agent 2,2’-pyridyl at 50 µM. When analyzed with the full promoter-reporter fusion construct (Psuf), luciferase reporter activity in the *sloR* mutant was reduced by >2-fold (P<0.01) when the strains were grown in BMG medium, especially in BMG with exclusion of Fe (BMG-Fe), as compared to the parent strain under the same conditions (**Figure 7**). Similar results were measured with the *sloR*/*perR* double mutant when grown in BMG, although no significant differences were measured when the double mutant was grown in BMG-Fe or BMG plus chelating agent (**Figure 7**).

To examine if SloR directly interacts with the promoter, regulating *sufCDSUB* expression, recombinant SloR (rSloR) was used in an EMSA assay with rSloR-*sloA* promoter interaction as a positive control (Spatafora et al., 2015). As expected, the inclusion of rSloR led to mobility shift of *sloA* probe. However, no apparent effect was observed when rSloR was mixed with the *sufCDSUB* probe on its gel mobility under the conditions studied (**Supplementary Figure 1**). Similar results were observed when *S. mutans* whole cell lysates were used in the EMSA assay. These results suggest that SloR is not directly involved in regulation of *sufCDSUB* expression, although it is also possible that other unknown factor(s) are involved in the SloR-mediated regulation, and in the absence of its co-factor, SloR will not bind to its promoter DNA in EMSA.

### The expression of *sufCDSUB* is induced by cysteine and requires SMU.852

**Figure 8:**
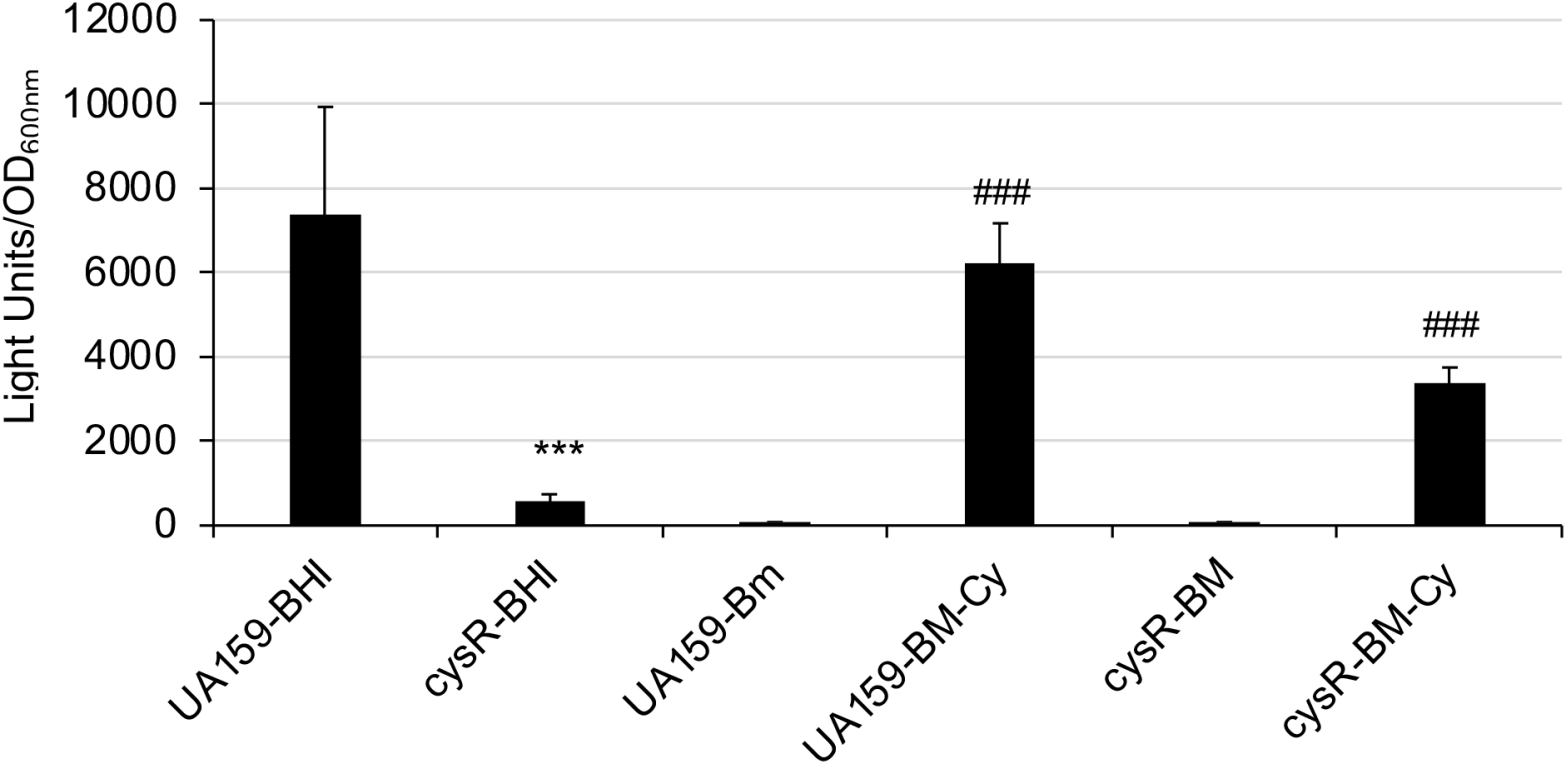
Luciferase reporter assays of a *cysR* mutant. *S. mutans* wild-type (UA159) and its cysR mutant (cysR) carrying the suf promoter-reporter were grown to mid-exponential phase in plain BHI (BHI) or modified BM (BM) medium with and without inclusion of cysteine (Cy, 4 mM). Results showed luciferase reporter expression under the direction of the sufCDSUB promoter was dramatically reduced in response to cysR deletion; and it was at its minimum when grown in BM medium without cysteine. ***, P<0.001, cysR vs UA159 in BHI; ###, P<0.001, BM-with cysteine vs BM without cysteine, when analyzed by Student *t*-test.

To test if cysteine also plays a direct role in SUF expression, the modified semi-defined biofilm medium with the omission of cysteine in the amino acid mix was used to grow the luciferase reporter strains, and the reporter activities were measured with and without addition of cysteine one hour prior to the luciferase assay. The results showed that reporter activity without cysteine was at its base line, while addition of cysteine at 4 mM was found to induce the reporter activity by 84.6 (± 7.4)-fold, compared to the ones receiving solvent control (P<0.001) (**Figure 8**). Addition of serine at 4 mM also led to increases of reporter activity, but to a much less degree with an average of 3.63(± 0.43)-fold (P<0.05). SMU.852, also *cysR* (Sperandio et al., 2010), encodes a LysR-family transcriptional regulator, which we have recently identified in a study of the regulation of *brpA* expression. To analyze if CysR plays a role in *sufCDSUB* expression, an allelic exchange mutant, TW660, was constructed, and relative to its parent strain, UA159, the *cysR* mutant displayed a reduced growth rate and optical density overnight when grown in regular BHI broth (**Supplementary Figure 2**). When transformed with the P*suf* promoter luciferase reporter fusion, the reporter activity in the *cysR* mutant was shown to be reduced by >11.06-fold, compared to the parent strain (P<0.001) (**Figure 8**). However, the reporter activity of the *cysR* mutant was still inducible with the addition of cysteine, when grown in the modified BM medium similarly as the wild-type, albeit to a reduced degree, which in part could be attributed to the reduced growth rate of the *cysR* mutant (**Supplementary Figure 2**). These results suggest that expression of *sufCDSUB* requires cysteine as an inducer, and CysR is a positive regulator of *sufCDSUB* expression.

**Figure 9:**
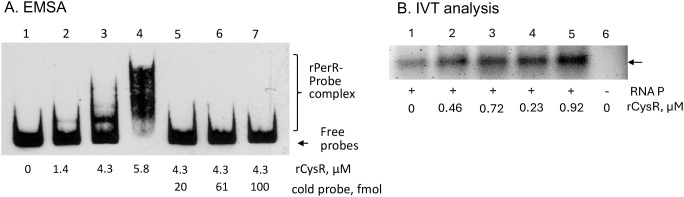
EMSA **(A)** and IVT **(B)** analysis of CysR. Inclusion of rCysR resulted in mobility retardation of biotinylated *suf* promoter DNA probes (Psuf, lanes 2-4), and such interaction was concentration-dependent as indicated. Addition of cold-probe led to release of biotinylated probes from the rPerR-promoter complex (lanes 5-7). **(B)** For IVT assay, inclusion of rCysR led to increase of *sufC* transcription (indicated by an arrow) under the direction of *suf* promoter. No RNA polymerase served as a negative control.

When analyzed, two regions with some similarities to the consensus of the LysR-type of regulators (https://regprecise.lbl.gov/sites.jsp?regulog_id=4369) were identified in the promoter region with one overlapping the -10 and the perR-box2 region and the other overlapping the Fur-Box1 site (**Supplementary Figure 3**). To test if CysR interacts with the *sufCDSUB* promoter, CysR was expressed and purified using affinity chromatography, and as shown in **Figure 9**, inclusion of the recombinant CysR protein (rCysR) was shown to cause mobility shift of the promoter probes, indicative of rCysR-promoter interaction. Consistent with the luciferase reporter fusion assays, rCysR was also shown in an IVT assay to enhance the transcription of *sufC* under the direction of *sufCDSUB* promoter (**Figure 9**). These results further support the role of CysR as a positive regulator of *sufCDSUB* in *S. mutans.*

## Discussion

Results presented here further demonstrated that consistent with our previous findings, the *sufCDSUB* operon in *S. mutans* is highly regulated in response to environmental cues and that multiple *cis-* and *trans-*acting factors have been shown to play a significant role in the regulation of *sufCDSUB* expression. These include transcription repressor PerR and Fe^2+^, as a co-effector, and positive regulator CysR. In addition, SpxA2, a global regulator in oxidative stress responses, is also required for *sufCDSUB* expression.

In *Synechocystis* spp. and several others, the expression of *suf* gene cluster is governed by *sufR*, which lies in front of the *suf* operon and acts as a repressor of the *suf* expression and an auto regulator of its own expression (Vuorijoki et al., 2017; Willemse et al., 2018; Cheng et al., 2020). Although *S. mutans* genome does not possess an apparent *sufR*, it contains a *perR* gene that encodes PerR, a peroxide stress regulator of the Fur family, widespread in many Gram-negative and low-GC Gram-positive bacteria such as *Bacillus* spp. and *S. pyogenes* (Bsat et al., 1998; Kajfasz et al., 2021). PerR is also iron-dependent, but unlike SufR, it does not directly interact with Fe-S clusters. Active PerR exists in homodimers, and each possesses two metal binding centers, with one for Zn^2+^required for conformation and dimerization and the other for Fe^2+^ or Mn^2+^ for promoter DNA interaction and regulation of target gene expression (Traore et al., 2006). However, recent studies have shown that in the presence of excessive iron (FeCl_2_) and upon exposure to hydrogen peroxide, the cysteine and histidine residues in the regulatory metal center can get oxidized quickly, which triggers a conformational change (Marinho et al., 2014; Lee and Helmann, 2006). Consequently, the regulator protein dissociates from the promoter, and the target gene gets transcribed. Like in group A streptococci, PerR in *S. mutans* has recently been shown by RNA-seq to be involved in regulation of a large amount of known genes including many involved in oxidative stress responses (Kajfasz et al., 2021; Ruxin et al., 2021). However, to the best of our knowledge, the current study is the first instance a direct link of PerR in regulation of *sufCDSUB* expression and Fe-S cluster homeostasis has been established.

Consistent with the role of PerR as a repressor in *sufCDSUB* expression, deletion of *perR* by allelic exchange led to significant increases of luciferase reporter activity, a de-repression of luciferase reporter expression. This is further supported by the result of site-directed mutations of a PerR-box adjacent to the extended -10 site-box, where EMSA showed that recombinant PerR interacts with the *sufCDSUB* promoter resulting in a mobility shift, and the result of IVT assay that the PerR-mediated repression is Fe^2+^-dependent. However, as mutations of selected nucleotides did not seem to significantly alter the PerR-promoter interaction, the exact nucleotides that interact with PerR await further investigation. It is also worth noting that PerR is known to be easily inactivated when exposed to iron and oxygen, weakening its capacity to bind to its target promoter, and such oxidation can be prevented by Zn^2+^ or Mn^2+^ (Lee and Helmann, 2006). Consistently, the inclusion of Zn^2+^ in the reaction mixture was shown to significantly enhance the rPerR binding and the consequent mobility shift in an EMSA.

Consistent with our previous finding that *sufCDSUB* expression is regulated in response to iron availability in the growth environment, cultures transferred from regular biofilm medium to medium with omission of FeCl_3_ or with inclusion of chelating agent 2,2’-pyridyl displayed >5-fold increases of luciferase reporter activity, but not in cells that were transferred from medium without inclusion of FeCl_3,_ indicative of iron as a co-repressor. The *in vitro* transcription assay leading to complete inhibition of suf transcription in the presence of recombinant PerR and Fe^2+^ corroborates the reporter activity findings. Iron is the second most abundant metal on earth, of which most exist in iron ions, Fe^2+^ and Fe^3+^. It is apparent that the amount of iron in the water and the basal medium components are sufficient for the bacterium to grow. It is the state of iron depletion that the bacterium senses from regular iron-rich condition to iron-limiting condition that triggers the up-regulation of the *sufCDSUB* expression. The incapability of the *perR* mutant to launch such a response to iron limitation is also consistent with the nature of its iron-dependence. The potential redox sensing ability that allows PerR-Fe^2+^ complexes to bind with the DNA and regulate gene expression of *sufCDSUB* needs further investigation.

Recent studies in group A streptococci and others have shown that PerR is a major oxidative stress-sensing repressor of H_2_O_2_ resistance (King et al., 2000; Ricci et al., 2002). Among other proteins and factors, PerR-deficiency leads to up-regulation of Dps-like peroxide resistance protein Dpr, a major H_2_O_2_ resistance factor. Dpr has been identified as a major factor in the resistance of *S. mutans* to ROS during growth in the presence of oxygen (Yamamoto et al., 2004). Intracellular free iron and hydrogen peroxide can oxidize a wide range of substrates and cause detrimental alterations of macromolecules and their related biological processes. The reaction, referred to as the Fenton reaction, is complex and capable of generating both hydroxyl radicals and higher oxidation states of the iron creating oxidative stress in the cell. Consistent with Fujishima et al. and Kajfasz et al (Fujishima et al., 2013; Kajfasz et al., 2021), deletion of *perR* in *S. mutans* led to up- and down-regulation of >35 proteins, including several sugar transporters and fructose polymer utilization. Of the up-regulated genes are those for major components of the oxidative stress tolerance pathways, including Dpr. Further study of *dpr* by allelic exchange mutagenesis further revealed that lacking of Dpr in *S. mutans* led to major defects in oxidative stress tolerance, significantly reduced its growth rate, and elevated autolysis of the *dpr* mutant, consistent with Kajfasz et al (Kajfasz et al., 2021). When compared to the wild-type, luciferase reporter activity in the Dpr-deficient mutant was elevated by >3-fold when grown in regular BHI, but no major differences were measured when grown in BHI broth plus methyl viologen at 2.5 mM (**Supplementary Figure 3**). The results suggest that unlike PerR, Dpr is a major factor that plays a direct role in oxidative stressor-mediated regulation of *sufCDSUB* expression in *S. mutans.*

Metalloregulator SloR is mostly known as a repressor to regulate the expression of target genes in response to manganese availability, including transporter SloABC for Mn^2+^/Fe^2+^in *S. mutans*. In *S. mutans*, it also positively regulates its own expression (Monette et al., 2018). However, unlike the repressor SloR, the binding site(s) for positive regulator SloR remains unclear and awaits further investigation. It is apparent that SloR-deficiency resulted in significant reduction of luciferase reporter activity, indicative of a potential role as a positive regulator in *sufCDSUB* expression, although EMSA showed no SloR-promoter DNA interaction under the condition studied. Considering the *sloR* deficient mutant also displayed growth defects when it was growing in Fe-rich medium, such growth defects could be in part attributed to the altered expression. However, it is also possible that other unknown factors are involved in SloR-mediated regulation of *sufCDSUB* expression. It is possible that SloR functions as a positive regulator, especially under iron depletion conditions, when PerR is mostly inactive, which also explains why in the presence of chelating agent the differences were not as significant, especially for the *sloR/perR* double mutant. Of note, the expression of SloR in *S. mutans* is also regulated by repressor PerR (Ruxin et al., 2021). Without SloR, the mutant can still take up the Fe in the culture medium, but its ability to assemble it into the Fe-S cluster is significantly reduced. Consequently, accumulation of free irons leads to the production of reactive oxygen species that causes cytotoxicity and growth defects including an extended lag phase. However, the exact role of SloR, as well as its binding site, in *sufCDSUB* regulation awaits further investigation. The results presented here also suggest that in *S. mutans*, repressor PerR and probably SloR, as a positive regulator, work in concert to regulate *sufCDSUB* expression most likely in response to the Fe-availability in the oral cavity, although how they coordinate in fine tuning the *sufCDSUB* expression remains unclear.

*S. mutans* possesses two global oxidative stress regulators, SpxA1 and SpA2, which have recently been shown by RNA-seq analysis to be required for expression of major oxidative stress genes including members of the sufCDSUB cluster (Galvao et al., 2015; Kajfasz et al., 2017). This study used luciferase reporter fusion assays and consistently, the results showed that the mutant lacking SpxA2, but not SpxA1, displayed major reductions in sufCDSUB expression. SpxA proteins are known to interact with the RNA polymerase in regulation of its target genes.

In Fe-S cluster assembly, cysteine is utilized by the desulfurase SufS to generate the sulfur for Fe-S cluster assembly (Lill, 2009; Sperandio et al., 2010). The result that addition of cysteine to the growth environment triggers dramatic increases of luciferase reporter activity suggests that the expression of *sufCDSUB* is regulated in response to cysteine as an inducer. A LysR family transcriptional regulator, CysR was previously found to play a role as an activator in regulating the expression of genes involved in cysteine biosynthesis and utilization in response to the availability of cysteine in the environment (Sperandio et al., 2010). In addition, it also indirectly regulates the expression of the genes involved in biosynthesis of methionine. An important amino acid, cysteine is also involved in various cellular processes, including protein synthesis and redox regulation. Methionine is an essential amino acid and serves as a methyl donor in various methylation reactions, playing a crucial role in epigenetic regulation and other cellular processes. It is therefore not surprising that the *cysR* mutant displays some major growth defects in growth rate and optical density. It is worth noting that no significant differences were observed in the wild-type for the growth rate and culture density between the cultures grown with and without inclusion of cysteine under the conditions studied. Like the wild-type, cysteine was able to induce the expression of the *sufCDSUB* gene cluster albeit to a less degree, which can be in part attributed to the reduced growth rate of the *cysR* mutant. The result also suggests that cysteine likely serves as a co-factor of CysR-mediated regulation of *sufCDSUB* expression.

In summary, the results presented here demonstrate that multiple factors work in concert to regulate *sufCDSUB* in *S. mutans* in response to iron and cysteine availability, other environment cues such as oxidative stressors, and other signals related to sulfur metabolism. These results also suggest that a keystone cariogenic bacterium, *S. mutans* inhabiting a unique niche in the oral cavity, may have evolved specific adaptations in its sole Fe-S cluster assembly machinery to cope with the challenges posed by its environment, such as fluctuations in pH, oxygen tension, and nutrient availability.

## Data Availability

The original contributions presented in the study are included in the article/**Supplementary Material**. Further inquiries can be directed to the corresponding author.
