# Supplementary material for: Multiple factors regulate the expression of sufCDSUB in Streptococcus mutans
Source: Front Cell Infect Microbiol. 2024 Nov 27;14:1499476. doi: 10.3389/fcimb.2024.1499476 (PMC11631912; doi:10.3389/fcimb.2024.1499476)
Supplement: Supplementary file 1 [file Image1.pdf]

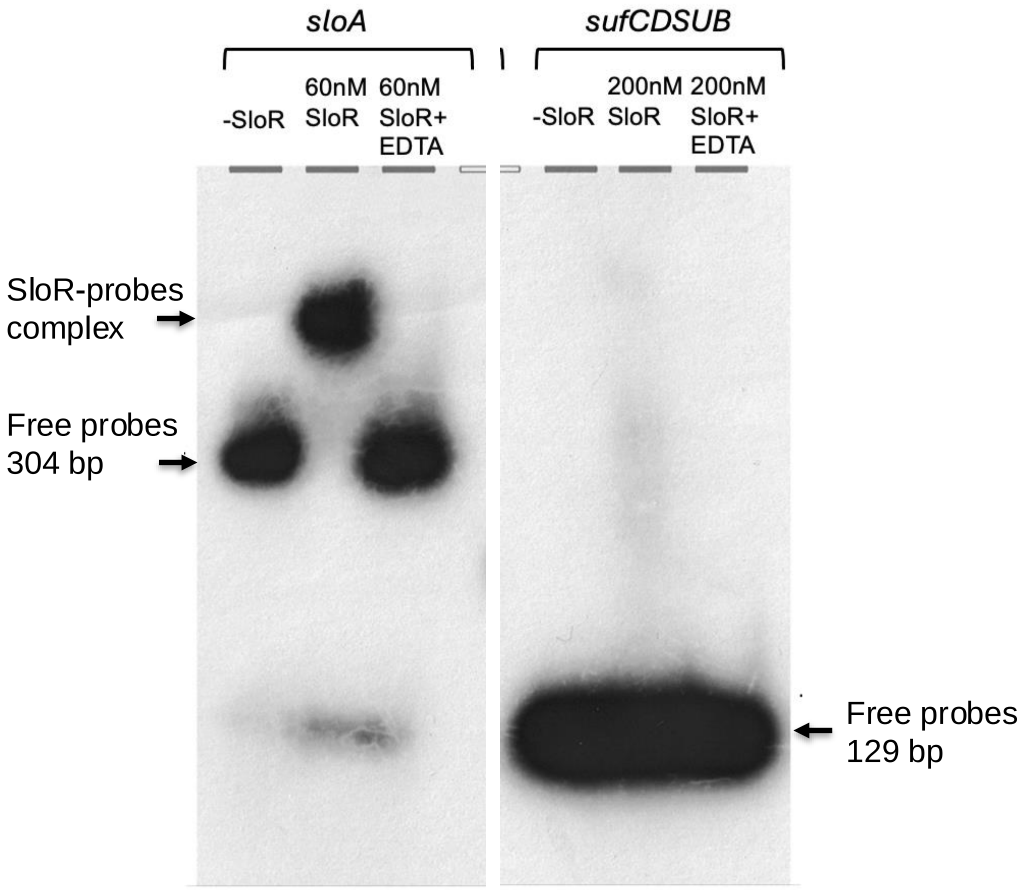

Figure S1. EMSA analysis of SloR in *sufCDSUB* expression. The *sufCDUSB* promoter probe was labelled with ^{32}P and mixed with recombinant SloR, and the protein-promoter probe interactions were separated by a 12% non-denaturing polyacrylamide gel (Spatafora et al., 2015). As illustrated by the left panel, inclusion of SloR at 60 nM led to mobility shift of the *sloA* promoter probe as a positive control (Spatafora et al., 2015), and addition of EDTA abrogated such an interaction. In contrast, no apparent probe shift was observed when SloR was included in the reaction with the *sufCDSUB* promoter probes, as shown in the right panel. Similar results were also obtained when *S. mutans* whole cell lysates were used in the reaction. The results were representatives of three repeats.

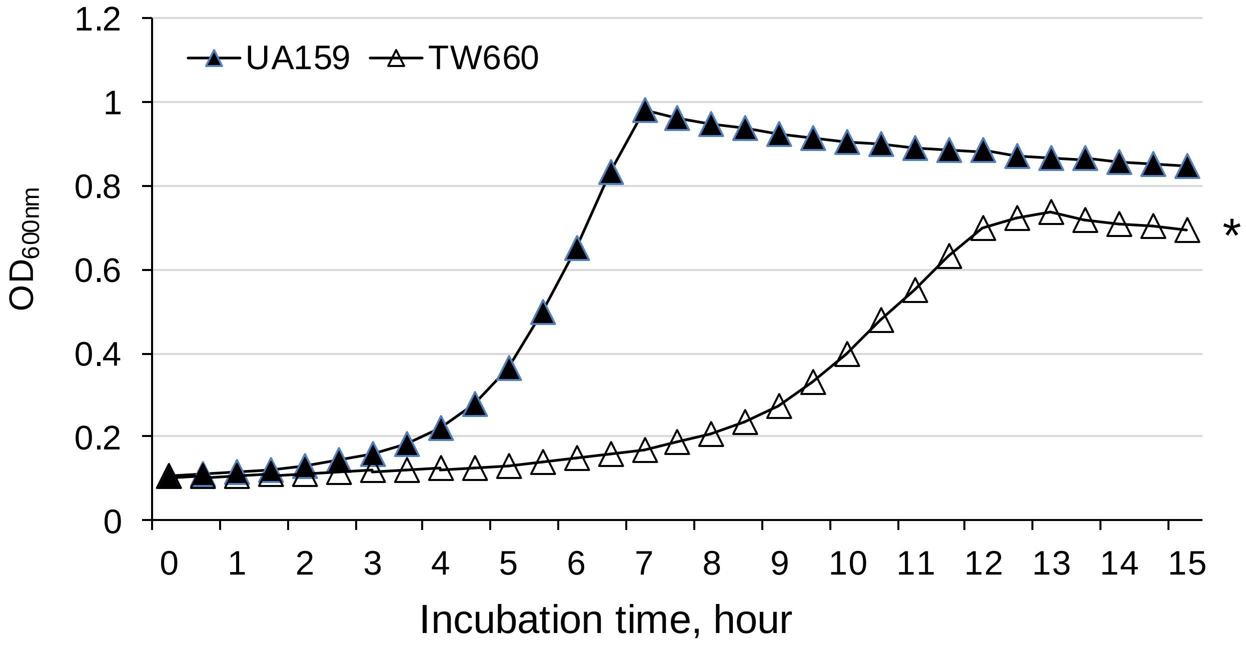

Figure S2. Growth characterization of a *cysR* mutant. *S. mutans* UA159 and its *cysR* mutant, TW660, were grown in plain BHI in a Bioscreen C. The results showed that the *cysR* mutant had a reduced growth rate and optical density overnight, as compared to its parent strain under the condition studied. *, *P*<0.05 vs UA159 when analyzed using Student *t*-test.

A. Consensus of CysR binding sites: https://regprecise.lbl.gov/sites.jsp?regulog_id=4369

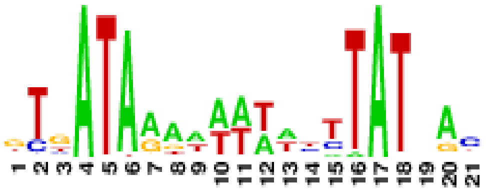

B. Pairwise alignment of the consensus of LysR with the promoter region of *sufCDSUB*:

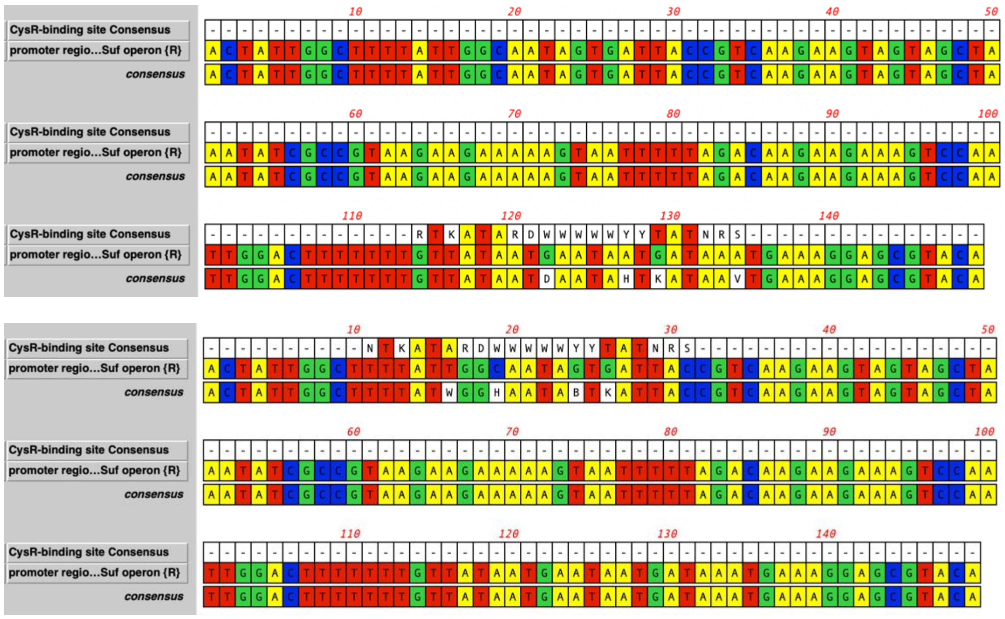

Figure S3. The consensus of the CysR binding site, as predicted (A) and identification of two regions in the *suf* promoter with similarity to the CysR-binding site by pairwise alignments (B).
