# Supplementary material for: Multiple factors regulate the expression of sufCDSUB in Streptococcus mutans
Source: Front Cell Infect Microbiol. 2024 Nov 27;14:1499476. doi: 10.3389/fcimb.2024.1499476 (PMC11631912; doi:10.3389/fcimb.2024.1499476)
Supplement: Supplementary file 3 [file DataSheet2.pdf]

Table S2. Proteins altered in the *perR* mutant*

Up-regulated proteins

Gene ID	Description	Ratio	P-Value
ccpA	catabolite control protein CcpA	1.376	0.02095
dpr	peroxide resistance protein Dpr	3.582	0.00006
glgA	glycogen synthase	1.800	0.00053
glgC	glucose-1-phosphate adenylyltransferase	2.133	0.00000
glgD	glycogen biosynthesis protein glgD	2.263	0.00934
scrA	PTS system sucrose-specific transporter subunit IIABC	1.492	0.04135
SMU_1410	reductase	3.041	0.03455
SMU_180	oxidoreductase	1.853	0.00024
SMU_1821c	aspartyl/glutamyl-tRNA amidotransferase subunit C	1.979	0.00018
SMU_1957c	PTS system mannose-specific transporter subunit IID	1.866	0.01634
SMU_1958c	PTS system mannose-specific transporter subunit IIC	1.799	0.01922
SMU_2127	succinate semialdehyde dehydrogenase	2.221	0.00009
SMU_447	hypothetical protein	1.330	0.04237
SMU_635	hypothetical protein, putative metal transporter	3.949	0.00000
SMU_636	N-acetylglucosamine-6-phosphate isomerase	2.438	0.00042
SMU_998	ABC transporter periplasmic ferrichrome-binding protein	1.341	0.00630

Down-regulated proteins

Gene ID	Description	Ratio	P-Value
fruA	exo-beta-D-fructosidase	0.648	0.00002
gtfB	glucosyltransferase-I	0.539	0.01319
lacD	tagatose 1,6-diphosphate aldolase	0.512	0.00227
mleS	malate dehydrogenase	0.638	0.03837
pfkB	fructose-1-phosphate kinase	0.744	0.01334
sloC	ABC transporter metal binding lipoprotein	0.793	0.02228
SMU_1442c	hypothetical protein	0.593	0.03618
SMU_1760c	hypothetical protein	0.614	0.00157
SMU_1764c	hypothetical protein	0.609	0.00523
SMU_205c	hypothetical protein	0.144	0.00006
SMU_209c	putative acetolactate synthase large subunit	0.138	0.00036
SMU_210c	putative acetolactate synthase small subunit	0.135	0.00081
SMU_609	40K cell wall protein	0.638	0.00049
SMU_689	hypothetical protein	0.757	0.04112
SMU_872	PTS system fructose-specific transporter subunit IIABC	0.740	0.00062
SMU.593	FurR-like regulatiry protein, PerR	0.112	0.04259
ssb2	single-stranded DNA-binding protein	0.282	0.00032

*****Data are presented in ratios of the perR mutant (PR) over the wild-type UA159 (UA).
